# First confirmed case of multisystem inflammatory syndrome in adult in Egypt

**DOI:** 10.1002/ccr3.7382

**Published:** 2023-05-31

**Authors:** Mohamed Abdel‐Salam Elgohary, Mohamed Emam Mohamed, Mahmoud Moustafa Hamada, Jackline S. Fahim, Thanaa A. El‐Masry, Ahmed Mohamed Ibrahim, Ahmad Gad Taha, Mohamed G. Seadawy, Athanasios Alexiou, Marios Papadakis, Gaber El‐Saber Batiha, Maisra M. El‐Bouseary

**Affiliations:** ^1^ Almaza Fever Hospital Cairo Egypt; ^2^ COVID Isolation Department Almaza Fever Hospital Cairo Egypt; ^3^ Microbiolgy Laboratory Department Almaza Fever Hospital Cairo Egypt; ^4^ Department of Pharmacology and Toxicology, Faculty of Pharmacy Tanta University Tanta Egypt; ^5^ Virology Laboratory Department Central Military Laboratories Cairo Egypt; ^6^ Biological Prevention Department Ministry of Defense Cairo Egypt; ^7^ Department of Science and Engineering Novel Global Community Educational Foundation Hebersham New South Wales Australia; ^8^ AFNP Med Wien Austria; ^9^ Department of Surgery II, University Hospital Witten‐Herdecke, Heusnerstrasse 40 University of Witten‐Herdecke Wuppertal Germany; ^10^ Department of Pharmacology and Therapeutics, Faculty of Veterinary Medicine Damanhour University Damanhour Egypt; ^11^ Department of Pharmaceutical Microbiology, Faculty of Pharmacy Tanta University Tanta Egypt

**Keywords:** COVID‐19, MIS‐A, MIS‐C, multiple organ dysfunctions, SARS‐CoV‐2 infection

## Abstract

**Key Clinical Message:**

Our case report demonstrates extremely uncommon data associated with MIS‐A, such as cholestatic jaundice, anemia, and quickly progressing pneumonia. IVIG and pulse steroid medications are the best treatments for improving clinical outcomes.

**Abstract:**

We report a case of multiple organ dysfunctions due to MIS‐A in an adult with a history of suspected COVID‐19. Our case demonstrates extremely uncommon data associated with MIS‐A, such as cholestatic jaundice, anemia, and quickly progressing pneumonia. IVIG and pulse steroid medications are the best treatments for improving clinical outcomes.

## 1 INTRODUCTION

In April 2020, initial reports from the United Kingdom documented eight pediatric cases with incomplete Kawasaki disease—like presentations.^1^ In May 2020, the Centers for Disease Control and Disease Prevention (CDC) termed it the multisystem inflammatory syndrome in children (MIS‐C) associated with COVID‐19. Since the CDC issued its initial MIS‐C definition, a lot has been revealed. A new case definition appeared on June 2022, which became effective for nonmandatory CDC reporting on January 1, 2023.^2^ In addition to Europe, Canada, the United States, and South Africa, there have also been reports of children who match this condition in China and other Asian nations.^3^

Several adult case reports have been published since June 2020 reporting this syndrome, which is known as “Multisystem Inflammatory Syndrome in Adults (MIS‐A).” MIS‐A is similar to MIS‐C and should be considered by clinicians and health departments.^4^, ^5^ Case reports of MIS‐A describe patients who had signs of SARS‐CoV‐2 infection that was confirmed by a PCR test and with minor respiratory symptoms but with severe inflammation confirmed by laboratory markers (e.g., elevated levels of C‐reactive protein “CRP,” ferritin, D‐dimer, cardiac enzymes, liver enzymes, creatinine) and multiple clinical symptoms, including fever and shock. Moreover, these patients showed symptoms of cardiovascular, gastrointestinal, dermatologic, and neurological illness.^5^

MIS‐A is likely underdiagnosed due to overlapping symptoms with severe COVID‐19, especially in patients with severe co‐morbidities. Its true pathophysiology is still a subject of debate.^6^ MIS‐A has so far predominantly been discussed in high‐income environments.^7^

The aim of the current study was to better comprehend and interpret MIS‐A in low‐ and middle‐income countries, where unique challenges exist between limited diagnostic and treatment options and the requirement to rule out a broader differential diagnosis that might resemble the features of MIS, such as severe/critical COVID‐19, classic Kawasaki syndrome, sepsis, and septic shock. Moreover, this report is a part of the global effort to establish a clear picture of this syndrome to enable further development of evidence‐based clinical guidelines to detect and treat affected patients early and properly.

## 2 CASE REPORT

A 22‐year‐old man was admitted to Almaza Fever Hospital, Cairo, Egypt, suffering from fever and mild upper respiratory symptoms 8 weeks prior with neither alarming basic laboratory investigations nor chest computed tomography (CT), which is routinely done on causality for fever cases. The patient has been vaccinated with 2 doses of the Sputnik‐V COVID‐19 vaccine, given 12 and 16 weeks prior, respectively. On March 10, he presented to the causality department with a fever of 38.5°C, a dry cough, and a sore throat for 1 day and was otherwise hemodynamically stable.

The patient was admitted to the ward, where basic laboratory investigations were performed. SARS‐CoV‐2 PCR swabs were requested as well as a chest CT (due to the current pandemic, doctors’ concern is usually directed toward the COVID‐19 infection). The summary of clinical presentations, laboratory measurements, and interventions over timeline of illness was presented in Table 1. All investigations and CT studies had nonsignificant results. Moreover, the COVID‐19 PCR test was negative. The patient received conservative treatment, including prophylactic antibiotic (ceftriaxone) and antipyretics, with no alarming new insult for the next 2 days except for an intermittent fever (≤39.5°C). At 4 a.m. on March 13th, the patient became drowsy, oliguric, and had unstable vital signs (GSC: 13/15; HR: 110 bpm; BP: 70/30 mmHg; RR: 45 breaths/min; SO2: 85% on room air; and body temperature: 39.5°C). Chest examination showed bilaterally reduced air entry with scattered coarse crepitation. He was transferred to the intensive care unit (ICU), where supplementary oxygen was given via a mask with a reservoir bag, alternating with a CPAP mask. A central venous line was inserted (CVP: 8 cm H_2_O). Norepinephrine (3.3 mg/kg/min) was used as a vasopressor, and IV fluids were guided by CVP measurement.

**TABLE 1:** Summary of clinical presentations, laboratory measurements, and interventions over timeline of illness.

Timeline	Clinical presentations/Laboratory measurements/Interventions
Preadmission	Mild symptomatic SARS‐CoV‐2 infection 8 weeks prior Fever, cough, and sore throat 1 day prior Chest CT and basic laboratory tests (CBC, creatinine, CRP, and liver enzymes) were normal
Admission Days 1 and 2	Negative SARS‐CoV‐2 PCR Conservative treatment for 2 days, with stable hemodynamic, intermittent fever (≤ 39.5°C), and persistent upper respiratory symptoms
Day 3	Drowsiness, dyspnea, oliguria, tachycardia, desaturation, and hypotension Electrocardiogram with sinus tachycardia Normal echocardiography and cardiac enzymes Rising white count, worsening renal function, elevated liver enzymes, leukocytosis, elevated inflammatory markers, elevated IL‐6, elevated D‐dimer, and elevated CK Bilateral pneumonia on chest CT Transferred to intensive care unit (ICU) with IV fluid, antibiotic, vasopressor, and oxygen supplementation via a mask with a reservoir bag, alternating with a CPAP mask Viral panels using PCR testing for upper respiratory viruses were negative, negative viral hepatitis screening, negative Quanteferon test, and auto‐immune screening Added Anakinera and methylpredisolone 2 mg/kg
Day 4	Improved conscious level, improved urine output, improved renal function, but still hypotensive Toxicology screening was negative Jaundice became clinically detectable Added pulse steroid and IVIG
Day 5	Anemia developed with normal blood film and negative Coombs test Marked thrombocytopenia with platelet transfusion
Days 6, 7, and 8	Improved hemodynamically and SO_2_ improved Decreasing the rate of vasopressor and using the simple oxygen mask Jaundice became unnoticed Blood, sputum, and urine culture were negative
Day 9	Patient developed polyuria with normal urine‐specific gravity SO_2_ was 94% on room air Desmopressin added to the treatment Improved chest CT (score 17/40)
Days 10 and 11	Norepinephrine was replaced with oral midodrine Urine output became normal MRI brain was normal
Days 12–15	Improved chest CT (score 5/40) Transferred to the ward on oral midodrine and oral prednisolone

The electrocardiogram (ECG) showed only sinus tachycardia. An echocardiogram was performed, which revealed mild mitral regurgitation. Abdomino‐pelvic U/S showed hepatomegaly and grade I nephropathy.

Emergency laboratory investigations showed hepatic and renal dysfunction, coagulopathy, leucocytosis, thrombocytopenia, increased inflammatory markers, a high D‐dimer level (1979/500 ng/mL), elevated total creatine kinase (CK) (2058/170), and an increased IL‐6 level (453 pg/mL), while CKMB was 72/16. The brain CT was irrelevant. Chest CT was evaluated and quantified using Yang et al.,^9^ which showed a severity score of 27/40 with bilateral scattered ground‐glass opacification/opacity (GGO) and consolidation of both the peribronchial and right lower lobes.

Testing for a variety of common upper respiratory pathogens such as Coronavirus 229E and HKUI; Rhinovirus/Enterovirus, Boca virus; *Mycoplasma pneumoniae*, *Bordetella pertussis*, Influenza A, A H1 and B and *Legionella pneumophila* was performed, and all of them came back negative. Pan‐cultures (blood, sputum, and urine cultures) were ordered on day 3 and revealed no growth on day 6. However, screening for COVID‐19 antibodies (IgM/IgG) was positive for IgG. Additionally, we performed auto‐immune screening (ANA, AMA, LKM, ASMA, and Rheumatoid Factor), screening for viral hepatitis (Anti‐HCV, Hbs Ag, Anti‐HBC IgM/IgG), testing of quantiferon—TB Gold and all were negative. Procalcitonin was nonspecific (0.08 ng/mL). The differential diagnosis was performed, including:

1. Severe COVID: studies and case reports on MIS‐A reveal that the disorder has a heterogeneous clinical presentation that frequently resembles severe COVID‐19. When PCR results are negative in patients with suspected acute COVID‐19, SARS‐CoV‐2 antibody testing (IgM antibodies) is pertinent for the diagnosis of acute COVID‐19‐related illness,^10^
2. Multisystem Inflammatory Syndrome in Adults (MIS‐A): According to the most recent CDC definition,^11^ patients under the age of 21 who are admitted to the hospital for more than 24 h or who experience a fatal illness should not have a more likely alternative diagnosis for the illness and must also comply with the specified clinical criteria and laboratory evidence,
3. Kawasaki disease: the diagnosis is established by a fever lasting five or more days. At least four of the following criteria should exist: polymorphous rash excluding bullous or vesicular eruptions, conjunctival injection, oropharyngeal mucous membrane changes, extremity changes, and lymphadenopathy. Symptoms common to MIS‐C but not typical of classic Kawasaki disease include abdominal pain, which is often more severe than in classic Kawasaki disease; thrombocytopenia, anemia, and lymphopenia; and elevated levels of ferritin, troponin, and D‐dimer,^12^
4. Septic shock: symptoms include fever, tachypnea, tachycardia, hypotension, and signs of tissue hypoperfusion. Its diagnosis is based on the recovery of the pathogen by culture.
5. Scarlet fever: rash, fever, and lymphadenopathy are present. A positive rapid streptococcal test or the culture result is diagnostic, and
6. Toxic shock syndrome: case definitions include hypotension and multisystem involvement in the presence of a history of retained foreign bodies (tampons and nasal packing material) and one or more sites of soft tissue necrosis in the case of *Staphylococcus aureus*, while the diagnosis is confirmed by the isolation of group A *Streptococcus* in the case of streptococcal toxic shock syndrome.

The criteria of our case were not consistently met with classic Kawasaki but were matched with MIS‐A. The reported case is a 22‐year‐old male who was admitted to the hospital with a fever that lasted for the first 3 days of hospitalization. The patient showed characteristics of MIS‐A, including conjunctivitis, skin rash, cardiovascular affections (tachycardia, severe hypotension, and shock despite normal echocardiography), hepatic and renal disorders (oliguria, and nephropathy),^13^ thrombocytopenia,^8^ increased inflammatory markers, increased IL‐6, and coagulopathy.^7^ Although there are yet no data from controlled clinical trials in adults with MIS‐A to support and guide the management of the syndrome, previous reports recommended the use of intravenous immunoglobulin, corticosteroids, or anti‐IL‐1 receptor antagonist chemotherapy.^5^

Anakinra (100 mg/0.67 mL) was given in a dose of 300 mg once daily intravenously for 4 days, followed by 100 mg once daily till clinically improved,^14^ totally for 5 days, methylprednisolone 2 mg/kg/d, remdesivir 200 mg initially and 100 mg/d for 5 days, and empiric antibiotics (levofloxacin 750 mg/d and meropenem 1 g TID), and fluconazole 200 mg/d. Prophylactic anticoagulation (fondaparinux 2.5 mg/d) was started. Within 1 day of initiating medication, the patient showed improved GCS to 15/15, his urine output improved, and his kidney function kept getting better. However, the vital signs did not seem to have improved.

On March 15, the patient received methylprednisolone 1 g/d and IV immunoglobulin 2 g/kg over 24 h of medication. The D‐dimer level was found to be 1979 ng/mL. There were no abnormalities in the blood film. The result of Coomb's test was negative. At 18000 plates, he received 12 packs or one platelet pack. The patient toxicological screening was unrestricted.

The patient started to improve clinically on March 16. The infusion rate of norepinephrine decreased while the blood pressure was elevated. As a result, both his oxygen consumption and saturation improved. On March 19, because of the patient's ongoing polyuria and normal urine‐specific gravity testing, desmopressin 120 mg biweekly was started. The oxygen supplementation was stopped when SO_2_ levels were above 94% in room air. Follow‐up on the chest CT. On March 20, the patient showed an improved CT‐SS (17/40). On March 21, the norepinephrine was replaced with midodrine 5 mg TID to support blood pressure. While taking desmopressin, the urine output began to decrease. An MRI of the brain was performed on March 24 in order to rule out central causes of polyuria, with unremarkable results. By the end of a couple of weeks, the patient was shifted to the ward, under oral desmopressin, prednisolone, and prophylactic apixaba. A follow‐up chest CT showed a CT‐SS of 5/40.

**FIGURE 1:**
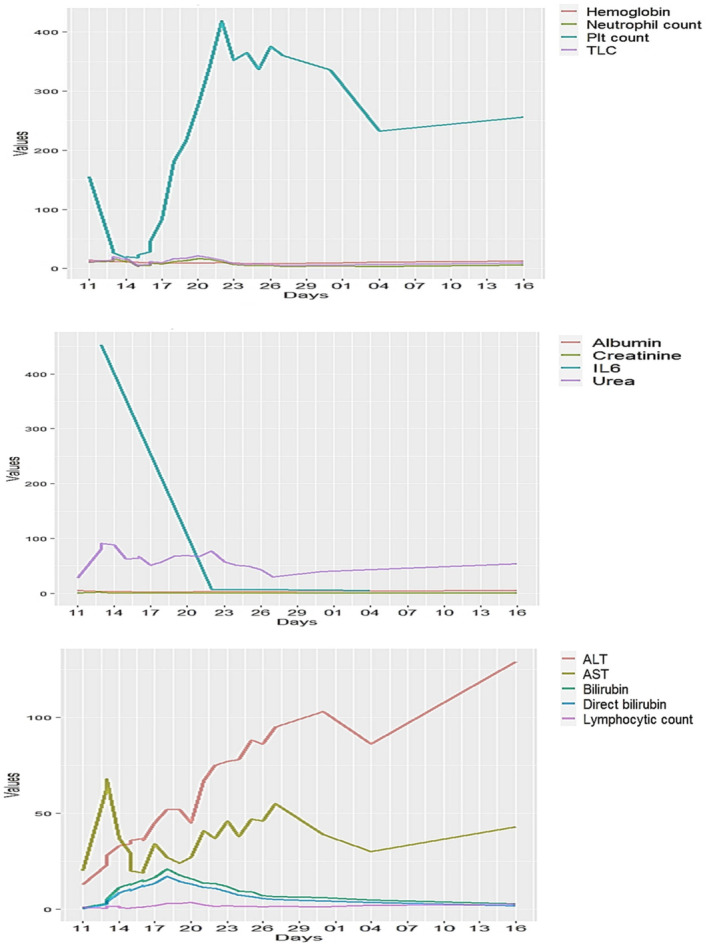
Line plots depicting the distribution of laboratory measurements over time (from March 11 to April 16).

**FIGURE 2:**
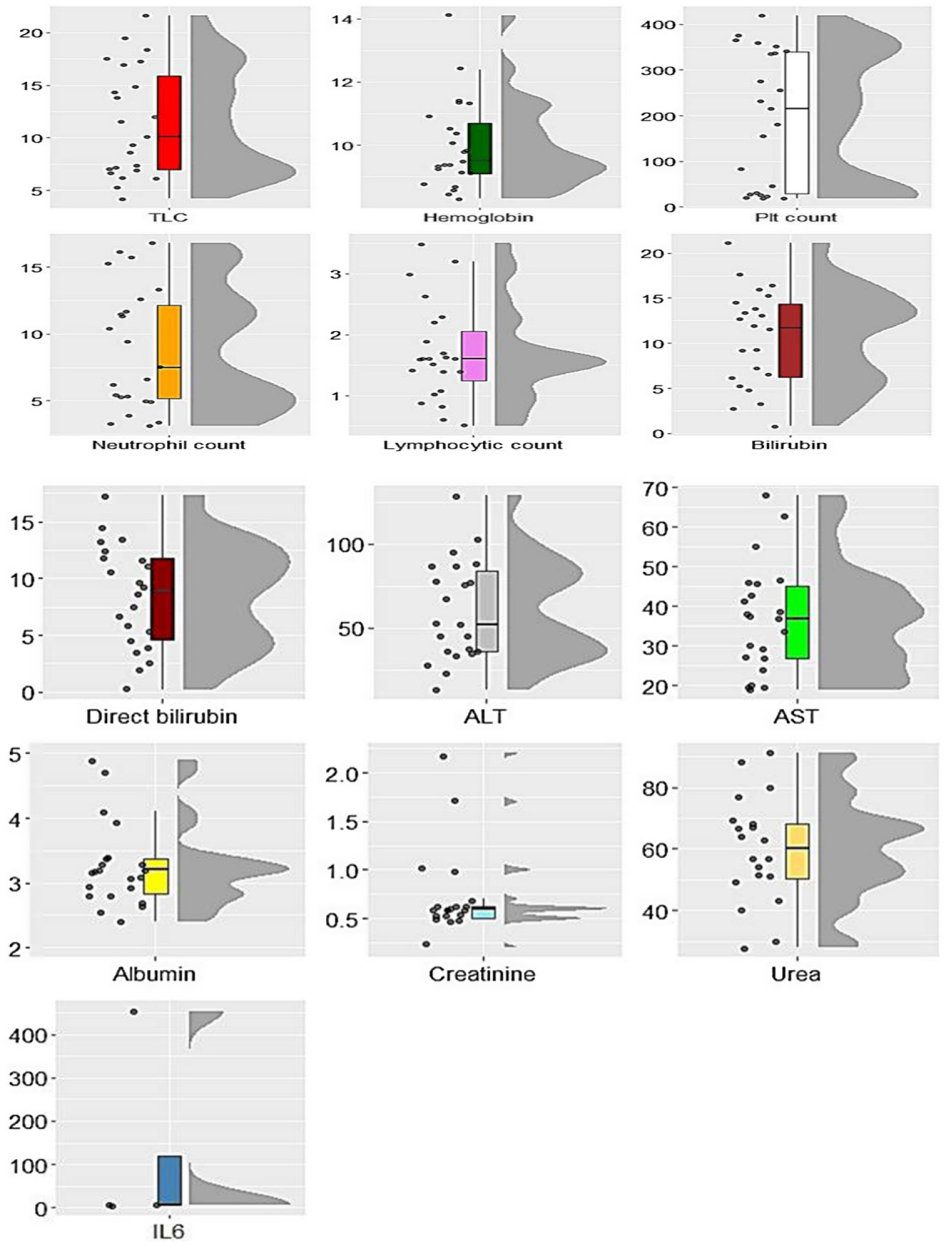
Raincloud plots and boxplots show the distribution of all laboratory measurements.

Figure 1 depicts the distribution of clinical laboratory measurements from 11 March to 16 April. IL‐6 measures were very high until 23/3 when there was a huge drop in IL‐6 to reach a value of 9 units. Urea measures reach 90 units on day 14/3 and start to decrease, reaching 30 units on day 25/3 and starting to increase a bit after that, hitting the peak of 60 units on 16/4. However, platelet counts decreased to reach 20 units on 14/3, increased again to more than 400 units on day 23/3, and started to decrease again to 260 units on 16/4. ALT measures hit a peak of 105 units on day 30/3 and decreased to reach 85 units on 5/4, then increased again to hit a peak of 130 units on 16/4. Also, AST measures increased to 70 units on day 12/3 and decreased again on day 16/3. After that, the AST measures hit another peak of 55 units and started to decrease again. Both bilirubin and direct bilirubin hit a peak of 23 units on day 18/3 and started to decrease again after that, till 5 units on day 16/4. The boxplots and the raincloud plots show the trends and the distribution of all 13 measurements taken in this case report as presented in Figure **2**.

## 3 DISCUSSION

MIS‐A is a severe hyper‐inflammatory syndrome that manifests with extra‐pulmonary multiorgans failure a few weeks just after the development of acute SARS‐CoV‐2 infections. The important constructive syndrome characteristics that were used for the syndrome case definition were the following^7^, ^8^, ^11^:

1. An increase in fever‐related inflammatory biomarkers (such as CRP, ferritin, d‐dimer, and so on),
2. Laboratory confirmation of COVID‐19 infection as a new infection (including RT‐PCR test positive and/or SARS CoV‐2‐positive antibody) approximately 12 weeks prior to onset,
3. Any infectious agents other than COVID‐19 should be excluded, and
4. To exclude the outcome of organ dysfunction and related tissue hypoxia, severe respiratory illness should not be present.

Besides the above criteria, at least two of the following essential characteristics should be included:

- Rash, plus or minus Conjunctivitis includes the nonpurulent type plus or minus mucocutaneous inflammation,
- Decreasing blood pressure plus or minus shock,
- Cardiac disorders that appear include valvulitis, myocarditis, or pericarditis. It also includes echocardiography abnormalities or increasing proBNP and troponin indicated in laboratory examinations.
- Coagulation defects are confirmed by clinical or laboratory test results and/or liver injury. The results included high D‐dimer, increased prothrombin time, and increased active partial thromboplastin time.
- Abdominal pain, vomiting, and/or diarrhea as gastrointestinal symptoms recently occurred.

Behzadi et al. investigated 36 instances of MIS‐A and concluded that the median patient age was 33 years old, 63% of the patients had no significant prior medical history, and 47% had SARS‐CoV‐2 infection, confirmed via PCR, antibody screening, or clinically. Fever was recorded in 86% of patients, while gastrointestinal symptoms were less frequently reported. A sore throat was observed in five patients (14%), and unilateral cervical pain or swelling was reported in four other cases (16%). In particular, tachycardia (61%) and hypotension/cardiogenic shock with verified reduced ejection fraction (64%) were reported as signs of cardiovascular failure.^12^

There is no agreement on the mechanism causing MIS‐A following the COVID‐19 infection. Several acute organ damages and systemic vasculitis are caused by MIS‐A, which is considered to be an extraordinary immune response. The significant improvement as a response to IVIG and aspirin confirms the existence of vasculitis.^12^ To determine how vasculitis and the SARS‐CoV‐2 infection are related, numerous theories have been proposed. For instance, the cytokine IL‐6, which also promotes vasculitis in Kawasaki disease, is one that increases significantly after COVID‐19 infection. IL‐6 renders lymphocytes more likely to adhere to endothelial cells, resulting in their damage.^15^ According to a different theory, immune complexes are deposited in capillaries, and complement is activated as the first insult.^16^ At the acute stage, it is regarded as a postinfectious condition instead of a primary infection.^15^, ^17^, ^18^ Moreover, numerous investigations have discovered that SARS‐CoV‐2 test results that are negative should not eliminate the infection.^18^, ^19^, ^20^

Several incidences of pulmonary affection in MIS‐A had been documented (pneumonia in 37%, shortness of breath in 52%).^8^ One MIS‐A report has provided an anemia description, and our reported case fits that description.^21^ Moreover, this is the first description of cholestatic jaundice in MIS‐A which has previously been documented in MIS‐C.^22^

In the present case, the Sputnik‐V COVID‐19 vaccine was administered twice, 2 months apart, and MIS‐A was discovered 3 months after the second dose. According to Ehikhametalor et al., MIS‐A emerged following a COVID‐19 infection and 3 weeks after the second dose of the vaccination. Further research is required to understand whether immunization may impact the COVID‐19 clinical picture.^10^ Besides that, Morataya et al. reported a case of MIS (MIS‐V) that manifested 2 days following the COVID‐19 vaccination and described seven additional cases of MIS‐V.^23^

After reviewing the literature, a large variety of management strategies and therapeutic approaches are suggested.^12^ In the current study, the patient received broad‐spectrum antibiotics, steroids, vasopressors, immunomodulators (anakinra), and IVIG treatment in addition to fluid resuscitation. Notwithstanding the variations in management, new research on the approaches for controlling MIS‐C has revealed no proof that IVIG alone or IVIG combined with steroids or immunomodulators results in greater rates of recovery.^8^

The current reported case is the third MIS‐A report from the Middle East, after the reported cases from Israel and the United Arab Emirates,^8^ and the second report from Africa after the report from South Africa.^7^ There are currently no definitive criteria for the diagnosis or treatment of MIS‐A because the existing research on the condition is still limited to tiny case studies and reports, despite the fact that it is expanding.^24^ Our study includes a list of learning points:

- MIS‐A is a significant fatal illness that can affect multiple organs. Our case demonstrates extremely uncommon data associated with MIS‐A, such as cholestatic jaundice, anemia, and quickly progressing pneumonia.
- Anti‐IL‐1 receptor antagonists, pulse corticosteroids, and IVIG have all been used to treat MIS‐A; these extremely effective treatments need to be further evaluated.

## 4 CONCLUSION

MIS‐A is a SARS‐CoV‐2 infection characterized by a delayed immunologic response and hyper‐inflammation. It should be suspected and identified by the clinical and public health communities using clinical evidence and empirical treatment to decrease morbidity and mortality outcomes. Moreover, the treatment should begin as soon as possible, with an accurate diagnosis and close monitoring. To avoid exacerbating the clinical condition, MIS‐A should be treated using the most effective methods. Anti‐IL‐1 receptor abtagonists, IVIG and pulse steroid medications are the best treatments for improving clinical outcomes. Although our presented case findings and management may not be broadly applicable to adults who may have MIS‐A, they do provide perspective on the difficulties in selecting a therapeutic option. Further research is needed to provide a comprehensive picture of this illness and to develop evidence‐based clinical guidelines. Long‐term follow‐up studies are necessary to investigate the potential consequences of MIS‐A.

## AUTHOR CONTRIBUTIONS

**Mohamed Abdel‐Salam Elgohary:** Conceptualization; data curation; investigation; visualization; writing – original draft; writing – review and editing. **Mohamed Emam Mohamed:** Data curation; investigation; writing – original draft. **Mahmoud Moustafa Hamada:** Data curation; investigation; writing – original draft. **Jaklin S. Fahim:** Data curation; investigation; writing – original draft. **Thanaa A. El‐Masry:** Investigation; visualization; writing – original draft. **Ahmed Mohamed Ibrahim:** Data curation; investigation; writing – original draft. **Ahmad Gad Taha:** Data curation; investigation; writing – original draft. **Mohamed G. Seadawy:** Conceptualization; data curation; investigation; visualization; writing – original draft; writing – review and editing. **Athanasios Alexiou:** Investigation; writing – original draft. **Marios Papadakis:** Investigation; writing – original draft. **Gaber El‐Saber Batiha:** Investigation; writing – original draft.

## FUNDING INFORMATION

The authors declare that no funds, grants, or other support were received during the preparation of this manuscript.

## CONFLICT OF INTEREST STATEMENT

The authors declare no competing interests.

## ETHICAL APPROVAL AND CONSENT TO PARTICIPATE

After informed written consent from the participant, the study has been approved by the Research Ethics Committee of the Faculty of Pharmacy, Tanta University (REC‐TP code: TP/RE/04/22p‐0034), and the ethical committee office of the Medical Military Academy in agreement with the Helsinki Declaration Roles.

## PATIENT PERCEPTION

The patient was delighted with the highly skilled medical service. He was surprised by the rapid response of the medical staff, and finally, the kind social support provided by the medical team.

## CONSENT

Written informed consent was obtained from the patient to publish this report in accordance with the journal's patient consent policy.

## Data Availability

All data generated or analyzed during this study are included in this published article.
